# Evaluation of the bacterial ocular surface microbiome in clinically normal horses before and after treatment with topical neomycin-polymyxin-bacitracin

**DOI:** 10.1371/journal.pone.0214877

**Published:** 2019-04-03

**Authors:** Erin M. Scott, Carolyn Arnold, Samantha Dowell, Jan S. Suchodolski

**Affiliations:** 1 Department of Small Animal Clinical Sciences, College of Veterinary Medicine & Biomedical Sciences, Texas A&M University, College Station, Texas, United States of America; 2 Department of Large Animal Clinical Sciences, College of Veterinary Medicine & Biomedical Sciences, Texas A&M University, College Station, Texas, United States of America; University of Minnesota Twin Cities, UNITED STATES

## Abstract

Next generation sequencing (NGS) studies have demonstrated a rich and diverse ocular surface-associated microbiota in people that was previously undetected by traditional culture-based methods. The ocular surface microbiome of horses has yet to be investigated using NGS techniques. This study aimed to determine the bacterial composition of the ocular surface microbiome in healthy horses, and to identify whether there are microbial community changes over time and following topical antibiotic use. One eye of 12 horses was treated 3 times daily for 1 week with neomycin-polymyxin-bacitracin ophthalmic ointment. Contralateral eyes served as untreated controls. The inferior conjunctival fornix of both eyes was sampled at baseline prior to initiating treatment (day 0), after 1 week of treatment (day 7), and 4 weeks after concluding treatment (day 35). Genomic DNA was extracted from ocular surface swabs and sequenced using primers that target the V4 region of bacterial 16S rRNA. At baseline, the most abundant phyla identified were Proteobacteria (46.1%), Firmicutes (24.6%), Actinobacteria (12.6%), and Bacteroidetes (11.2%). The most abundant families included Pasteurellaceae (13.7%), Sphingomonadaceae (7.9%), an unclassified Order of Cardiobacteriales (7.7%), and Moraxellaceae (4.8%). Alpha and beta diversity measurements were unchanged in both treatment and control eyes over time. Overall, the major bacterial taxa on the equine ocular surface remained stable over time and following topical antibiotic therapy.

## Introduction

The ocular surface microbiota refers to the resident microorganisms that colonize the cornea, conjunctiva, and tear film. The equine ocular surface is prone to developing serious, vision-threatening ocular diseases such as infectious ulcerative keratitis, and is often treated with topical broad-spectrum antibiotics such as neomycin-polymyxin-bacitracin [1–4]. Evidence suggests that ocular surface microbiota play a protective role in preventing the proliferation of pathogenic species and thus, changes in the microbiome may be linked to ocular diseases [5,6]. Additionally, external factors, such as short or long-term use of topical antibiotics may influence the composition and stability of microbial communities [7].

Traditionally, bacterial microorganisms on the ocular surface of both healthy and diseased horses have been studied using conventional culture-based techniques [1–4,8–12]. Gram-positive bacteria were reported to predominate the equine ocular surface, with *Bacillus*, *Staphylococcus*, *Streptococcus*, and *Corynebacterium spp*. commonly cultured regardless of geography, climate, or season [1–4,8–12]. Less frequent isolates included gram-negative bacterial microbes such as *Moraxella*, *Acinetobacter*, *Neisseria* and *Pseudomonas spp*. [1–4,8–12]. Very few culture-based studies have evaluated changes in the ocular surface microbiota over time or following topical antibiotic use [4,10]. These limited studies identified a relatively stable community of bacterial organisms with no significant effect of time [4] or antibiotic use [10] on the frequency or type of bacterial isolates cultured.

Overall, the ocular surface is not abundantly colonized by bacteria, with frequent reports of no growth from samples of healthy horse eyes [2, 9–12]. This finding highlights the limitations of culture-based methods, as many bacterial organisms do not grow well in laboratory conditions and thus cannot be readily identified in a given sample. The advent of molecular-based methods, such as 16S rRNA sequencing, have allowed for more extensive and detailed identification of the species composition of the ocular surface microbiota in humans [13–17], cats [18] and dogs [19]. Preliminary studies have revealed an unexpectedly diverse and distinct microbial community on the ocular surface compared to culture-based studies [13–19].

Presently, there are no published reports evaluating the ocular surface microbiome of horses using molecular-based techniques. Such investigations may one day lead to an improved understanding of ocular diseases in both veterinary and physician ophthalmology. This study aimed to determine the bacterial composition of the ocular surface microbiome in healthy horses, and to identify changes in the microbial community over time and following topical antibiotic use.

## Materials and methods

### Participants

The study was approved by the Texas A&M University Institutional Animal Care and Use Committee (Animal Use Protocol #2017–0333). Twelve horses, free of ocular disease, were selected from a teaching herd at the Department of Large Animal Clinical Sciences at Texas A&M University College of Veterinary Medicine & Biomedical Sciences. Included were five mares and seven stallions with ages ranging from 7–25 years (Table 1). The study was performed in December and January in east-central Texas. The average temperature throughout the study period was 56 degrees F (Min: 44; Max: 67) with an average humidity of 70%. The mares were pastured throughout most of the year and housed in individual indoor stalls throughout the study. Stallions were housed year-round in individual stalls within an open-air pavilion. Horses were provided with free-choice water and hay, and were fed grain daily.

**Table 1:** Study population: Signalment and randomization of eyes for healthy horses.

Horse	Breed	Age (Y)	Sex	Treatment Eye	Control Eye
**1**	Quarter Horse	16	F	OS	OD
**2**	Quarter Horse	14	F	OD	OS
**3**	Quarter Horse	17	F	OD	OS
**4**	Quarter Horse	9	F	OD	OS
**5**	Quarter Horse	11	F	OS	OD
**6**	Quarter Horse	17	M	OS	OD
**7**	Quarter Horse / Morgan cross	22	M	OD	OS
**8**	Arabian	25	M	OS	OD
**9**	Quarter Horse	17	M	OS	OD
**10**	Quarter Horse	7	M	OS	OD
**11**	Quarter Horse	17	M	OD	OS
**12**	Thoroughbred Horse	14	M	OD	OS

Abbreviations: Y: years, F: female (mare), M: male (stallion), OS: left eye, OD: right eye.

## Sample collection

All horses had a complete ophthalmic examination performed by a board-certified veterinary ophthalmologist (EMS). This included evaluation of the anterior segment of the eye by slit-lamp biomicroscopy (SL-17, Kowa Optimed Inc., Torrance, CA), and the posterior segment of the eye by indirect ophthalmoscopy (Vantage Plus Wireless Headset, Keeler Instruments Inc., Malvern, PA). A routine minimal ophthalmic database was also performed. This included Schirmer tear test measurements (Intervet Inc., Summit, NJ), fluorescein staining (Amcon Laboratories Inc., St. Louis, MO), and tonometry (Tono-Pen, Dan Scott and Associates, Inc., Westerville, OH). Any horse with an abnormal ophthalmic exam or minimal database result was excluded from the study.

Baseline conjunctival swab samples were collected after the Schirmer tear test and before fluorescein staining and tonometry in order to prevent contamination or dilution of the sample. A volume of 0.2 ml 0.5% tetracaine (Bausch & Lomb Inc., Tampa, FL) was placed on the ocular surface of each eye to provide topical analgesia. The inferior conjunctival fornix of both eyes was sampled with Isohelix buccal swabs (Boca Scientific, Inc. Westwood, MA). Two swabs were used per each site, and each side of the swab was rubbed in the conjunctival fornix 10 times. The swabs were collected in DNeasy Powerbead tubes with 750-μl buffer containing guanidine thiocyanate (QIAGEN, Inc., Germantown, MD). A volume of 0.2 ml 0.5% tetracaine was placed on a third swab at the same time and place of subject testing to serve as a negative control to confirm a lack of environmental contamination. All samples were immediately stored at 4 degrees C for no longer than 24 hours until the extractions were performed.

Once baseline samples were collected, one eye of each horse was randomly selected for treatment with a topical broad-spectrum triple antibiotic ointment, neomycin-polymyxin B-bacitracin (Dechra Veterinary Products, Overland Park, KS). Randomization of eyes into treatment and control groups for each horse was determined using online software (https://www.randomizer.org). One half-inch strip of triple antibiotic ointment was applied directly to the ocular surface of the selected eye of each horse three times daily for 7 days. Handlers wore nitrile exam gloves while administering the ophthalmic medication. Repeat conjunctival swabs occurred at the completion of topical antimicrobial therapy and 4 weeks after therapy ended.

### DNA extraction and sequencing

Genomic DNA was extracted from the swabs using the DNeasy Powersoil DNA isolation kit (QIAGEN, Inc., Germantown, MD) following the manufacturer’s instructions. Negative controls consisting of unused swabs and 0.2 ml 0.5% tetracaine were processed through DNA extraction and verified to contain <1% of total OTUs for all bacterial taxa.

Sequencing of the 16S rRNA gene V4 variable region was performed at MR DNA Laboratory (www.mrdnalab.com, Shallowater, TX, USA) on an Illumina MiSeq platform (Illumina Inc., San Diego, CA) to produce 2x300 paired-end reads using 515F (5’ -GTGYCAGCMGCCGCGGTAA- 3’) and 806R (5´-GGACTACNVGGGTWTCTAAT- 3´) primers.

### Data analysis

The software Quantitative Insights Into Microbial Ecology (QIIME 2) was used for processing and analysis of sequences [20]. Raw sequences were de-multiplexed and low quality reads were filtered using default parameters for QIIME. Chimeric sequences were detected using DADA2 and removed prior to analysis [21]. Operational taxonomic units (OTUs) were assigned and clustered using an open-reference OTU picking protocol in QIIME and defined as having 97% similarity to the Greengenes database [22,23]. Next, contaminant sequences determined to be mitochondria, chloroplasts, unassigned, or those associated with the phylum cyanobacterium, were excluded from further analysis. Data were deposited in the National Center for Biotechnology Information (NCBI) Sequence Read Archive (SRA) under the accession number SRP161476.

Alpha diversity was calculated using observed OTUs, Shannon, and Chao1 metrics to compare species richness and evenness between control and treatment eyes at baseline and among control and treatment eyes over time. Statistical analysis of alpha diversity indices was performed using the software package PRISM (PRISM 7, GraphPad Software Inc., San Diego, CA). Since data were assumed to follow a non-normal distribution, a non-parametric Wilcoxon matched-pairs signed-ranks test was used for statistical comparison between treatment and control eyes at baseline. A non-parametric Friedman test, followed by a Dunn’s multiple comparison post-test were performed to assess differences in treatment and control eyes over three time points [24].

Beta diversity (bacterial community composition) was calculated using both weighted and unweighted UniFrac metrics to measure similarity between samples, and visualized for clustering with Principle Coordinate Analysis (PCoA) plots. An Analysis of Similarity test (ANOSIM) within PRIMER 6 (PRIMER-E Ltd. Luton, UK) software package was performed on the beta diversity distance matrices to assess differences in bacterial community composition between samples. Microbial communities compared by ANOSIM have an R statistic near 1 when they are different and near 0 when they are similar in composition.

Differences in the relative abundance of bacterial taxa between eyes at baseline, and among control and treatment eyes over time, were investigated. Data were tested for normality using the Shapiro-Wilk test and most datasets did not meet the assumptions of normality (JMP Pro 14, SAS, Marlow, Buckinghamshire). Therefore, a non-parametric Mann-Whitney U test was used for statistical comparison between treatment and control eyes at baseline. A non-parametric Friedman test was performed to assess differences in treatment and control eyes over three time points (PRISM 7, GraphPad Software Inc., San Diego, CA). A Dunn’s multiple comparison post-test was then used to determine which time points were significantly different. P-values were adjusted for multiple comparisons and corrected for false discovery rate [24]. P- and q-values <0.05 were considered statistically significant.

Linear discriminant analysis effect size (LEfSe) was performed using Calypso to analyze the abundance of bacterial taxa in treatment and control eyes and their associations with each time point [25, 26].

## Results

### Sequence analysis

All sequences were rarified to an even sequencing depth of 9,610 sequences per sample to correct for unevenness between samples. Samples were collected from 24 eyes at three time points for a total of 72 samples. Negative control samples were not included in data analysis as they did not contain suspected contaminants from sampling or PCR amplification. A total of 3,878,314 sequences were amplified (Min: 9,618; Max: 232,220; Median: 41,419; Mean: 52,865; SD: 41,004). Data were used to define the relative abundance of bacteria for each individual sample.

### Healthy horse eyes at baseline

#### Species richness and diversity

**Fig 1:**
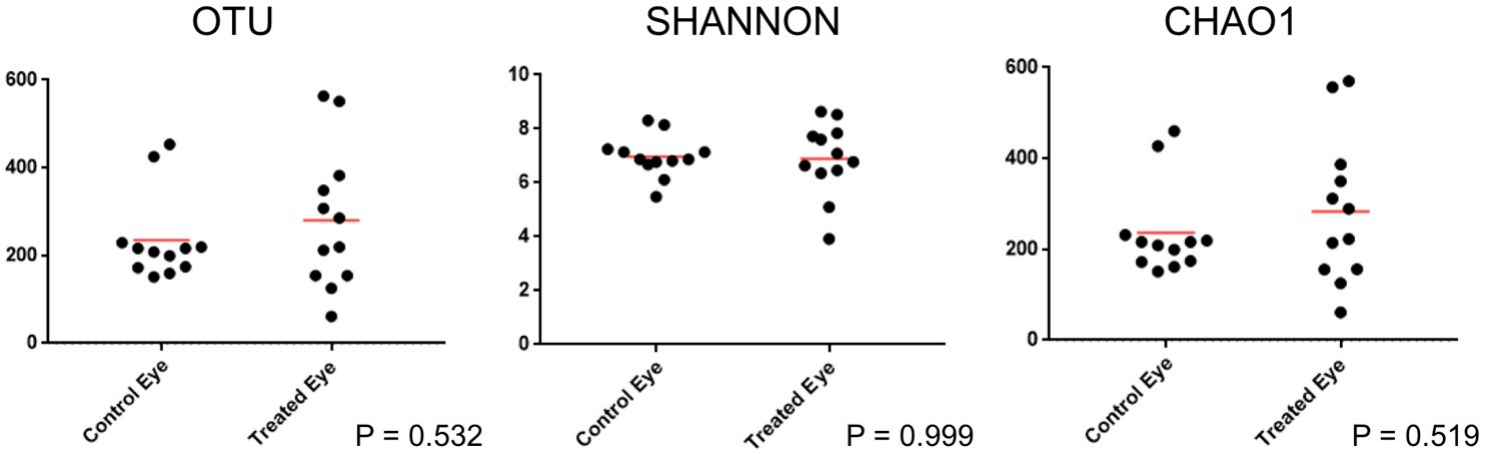
Scatter plots and statistical evaluation of 16S rRNA gene sequences obtained from 12 healthy horses (24 eyes), comparing treatment and control eyes at baseline (day 0). Each dot represents one eye. There is no difference in alpha diversity between eyes at baseline (Wilcoxon match-pairs signed-ranks test).

Baseline samples from treatment and control eyes were compared prior to antibiotic treatment (Table 2). The three alpha diversity metrics used included observed OTUs, which provides insight into the richness of the microbial communities present, Shannon, which considers both abundance and evenness, and Chao1, which estimates richness at full sequencing coverage. Wilcoxon match-pairs signed-ranks test revealed no significant difference between control eyes and treatment eyes at baseline for all three alpha diversity metrics. Hence, there was no difference in species richness, evenness, or abundance between eyes at baseline (Fig 1).

**Table 2:** Summary of alpha diversity indices at a depth of 9,610 sequences per sample for control and treatment eyes at baseline.

	Control Eyes	Treatment Eyes	*P-value
**Observed OTUs**	235.1 ± 98.7	283.2 ± 152.0	0.532
**Shannon**	7.0 ± 0.8	6.9 ± 1.4	0.999
**Chao1**	236.0 ± 100.4	283.2 ± 162.0	0.519

Values represent averages with standard deviations.

*P-values determined by Wilcoxon matched-pairs signed-ranks test with significance level < 0.05.

#### Microbial community structure

**Fig 2:**
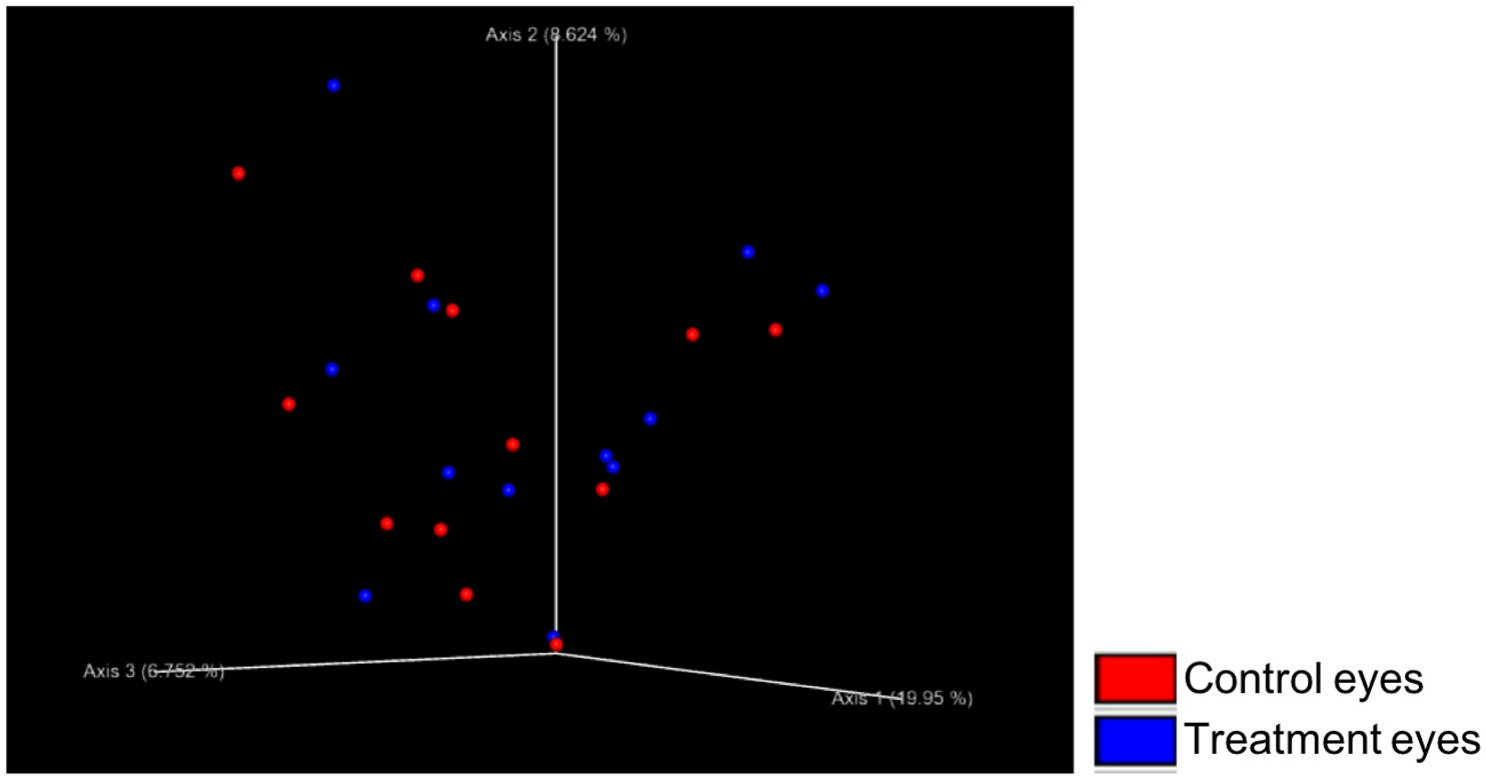
Principle coordinate analysis plot (PCoA) of unweighted UniFrac distance matrices between treatment and control eyes at baseline (day 0). Each dot represents the microbial composition of one eye. No clustering was observed indicating there was no difference in beta diversity between eyes at baseline.

Beta diversity measures (weighted UniFrac, unweighted UniFrac) indicated there was no significant difference in community structure between treatment and control eyes at baseline (R = -0.074, R = -0.029, respectively, p > 0.05). Control eyes did not cluster differently from treatment eyes at baseline (Fig 2).

#### Microbial community composition

**Fig 3:**
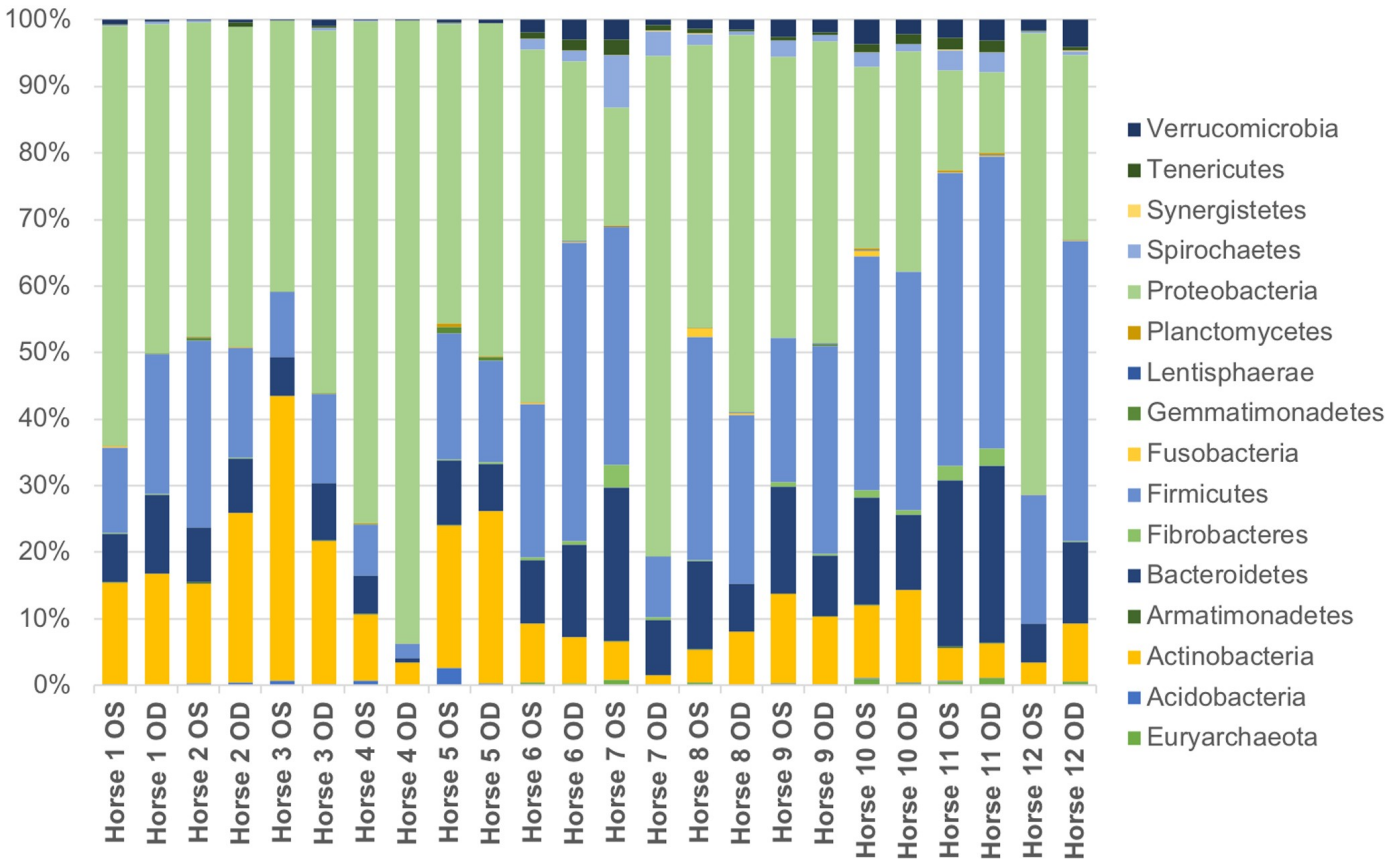
Composition of the ocular surface microbiome in healthy horses. Relative abundance of taxa annotated to the level of bacterial phylum at baseline (day 0). Each bar chart represents the left (OS) or right (OD) eyes of 12 horses.

Using a Mann-Whitney U test, we found there was no significance difference in bacterial taxa abundance between treatment and control eyes at baseline. Data from all 24 eyes were averaged to describe the bacterial taxa composition of the healthy equine ocular surface. A total of 17 bacterial phyla were detected and 5 phyla were present in all 24 eyes (Table 3). The most common phyla were Proteobacteria (46.1%), followed by Firmicutes (24.6%), Actinobacteria (12.6%), and Bacteroidetes (11.2%) (Fig 3).

**Table 3:** Taxa present at ≥ 1% mean relative abundance in healthy horses at baseline. Mean relative percentages and standard deviation of the most abundant bacterial groups, annotated to the level of phylum, family, and genus, based on sequencing of the 16S rRNA.

Taxon	Healthy Horses at Baseline		
Phylum -Family --*Genus*	Mean %	SD %	Number of eyes with positive detection (n = 24)
**Proteobacteria**	46.1	20.0	24
-Pasteurellaceae	13.7	18.8	24
-Sphingomonadaceae	7.9	5.0	24
--*Sphingomonas spp*.	7.2	4.8	24
-Unclassified Cardiobacteriales	7.7	20.0	24
-Moraxellaceae	4.8	4.1	24
--*Acinetobacter spp*.	2.3	2.2	22
--*Moraxella spp*.	1.6	1.8	23
-Pseudomonadaceae	1.5	1.0	24
--*Pseudomonas spp*.	1.5	1.0	24
-Aurantimonadaceae	1.3	0.9	22
-Methylobacteriaceae	1.3	0.9	23
--*Methylobacterium spp*.	1.0	1.0	23
-Rhizobiaceae	1.0	1.0	22
--*Agrobacterium spp*.	1.0	0.5	20
-Comamonadaceae	1.0	1.0	21
**Firmicutes**	24.6	12.9	24
-Ruminococcaceae	4.5	4.5	23
-Gemellaceae	4.1	4.4	24
-Lachnospiraceae	3.1	2.6	24
-Unclassified Clostridiales	2.4	2.2	22
-Planococcaceae	1.7	3.3	14
-Streptococcaceae	1.3	1.3	22
--*Streptococcus spp*.	1.3	1.3	22
-Erysipelotrichaceae	1.3	1.0	21
--*RFN20 spp*.	1.0	1.0	19
-Bacillaceae	1.1	1.2	23
--*Bacillus spp*.	1.0	1.0	15
-Staphylococcaceae	1.0	1.1	22
--*Staphylococcus spp*.	1.0	1.0	19
-Clostridiaceae	1.0	0.5	23
**Actinobacteria**	12.6	9.3	24
-Corynebacteriaceae	2.6	4.8	22
--*Corynebacterium spp*.	2.6	4.8	22
-Microbacteriaceae	2.4	1.6	24
-Micrococcaceae	2.1	2.0	22
--*Arthrobacter spp*.	1.5	2.2	11
-Brevibacteriaceae	1.0	1.0	19
--*Brevibacterium spp*.	1.0	1.0	19
-Gordoniaceae	1.0	1.0	20
--*Gordonia spp*.	1.0	1.0	20
-Nocardioidaceae	1.0	1.0	21
**Bacteroidetes**	11.2	6.3	24
-Unclassified Bacteroidales	5.0	5.1	23
-Sphingobacteriaceae	1.3	1.7	24
-Cytophagaceae	1.1	1.0	24
--*Hymenobacter spp*.	1.0	1.0	22
-RF16	1.0	1.0	19
-[Paraprevotellaceae]	1.0	1.0	22
**Verrucomicrobia**	1.5	1.2	24
-RFP12	1.4	1.2	21
**Spirochaetes**	1.3	1.7	20
-Spirochaetaceae	1.2	1.7	20
--*Treponema spp*.	1.2	1.6	20

**Fig 4:**
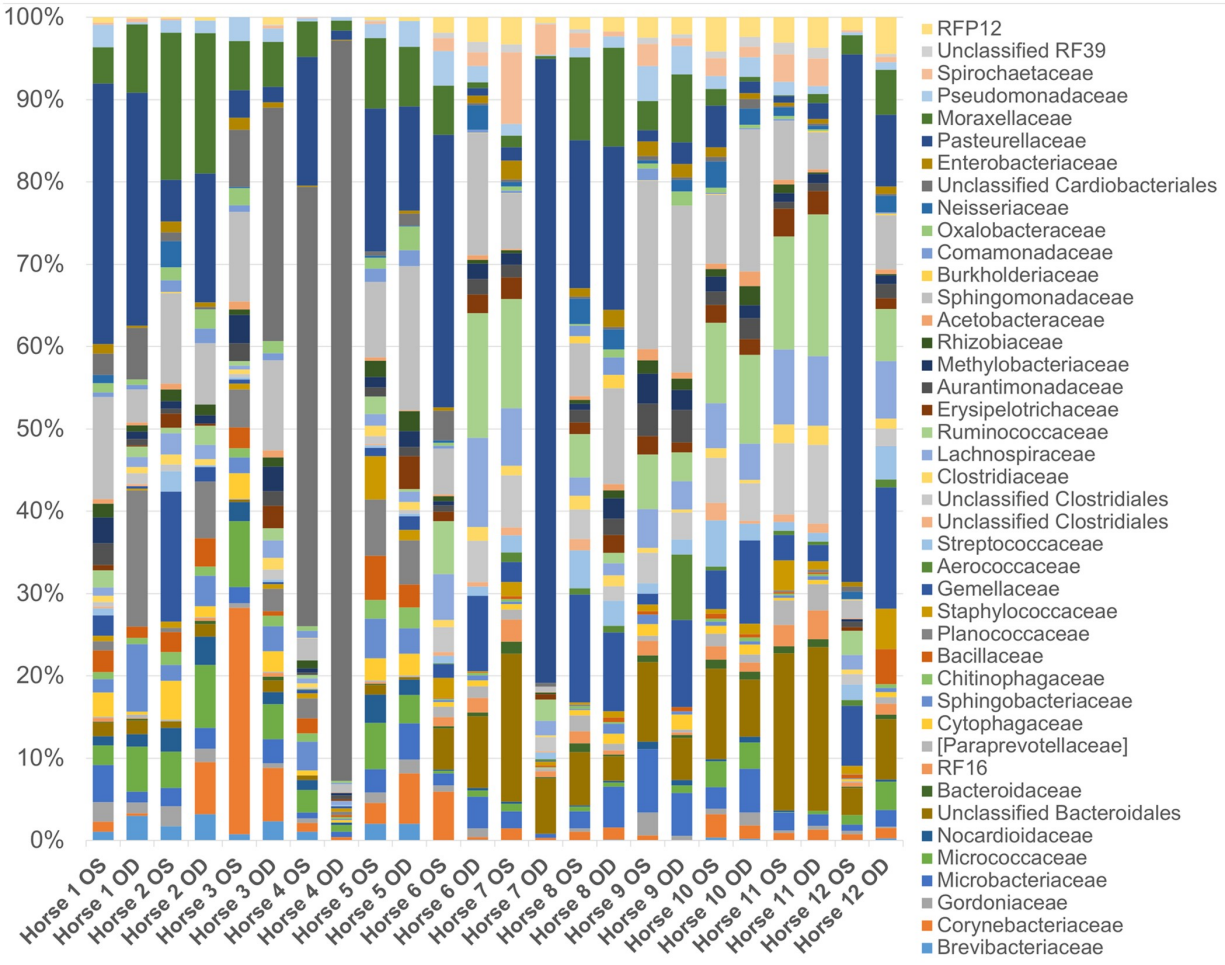
Composition of the ocular surface microbiome in healthy horses. Relative abundance of taxa annotated to the level of bacterial family at baseline (day 0). Each bar chart represents the left (OS) or right (OD) eyes of 12 horses.

A total of 52 bacterial families were detected and 10 families were present in all 24 eyes (Table 3). The most common bacterial families sequenced were Pasteurellaceae (13.7%), Sphingomonadaceae (7.9%), and an unclassified order of Cardiobacteriales (7.7%). Other commonly identified families present in most eyes included an unclassified order of Bacteroidales (5%), Moraxellaceae (4.8%), Ruminococcaceae (4.5%), and Gamellaceae (4.1%) (Fig 4). Bacterial families commonly cultured from the equine ocular surface, Corynebacteriaceae, Streptococcaceae, Bacillaceae and Staphylococcaceae, represented 2.6%, 1.3%, 1.1% and 1.0% of the bacterial families sequenced, respectively. At the phylum and family levels, there was individual variation in the relative abundances of bacterial taxa both between eyes and between horses; however, the overall composition remained consistent (Figs 3 and 4). Throughout all samples, an average of 259 different OTUs were detected.

### Temporal variability of ocular surface microbiome in control eyes

In order to understand the temporal stability of the ocular surface microbiome in healthy horses, two additional samples were collected from control eyes one week (day 7) and five weeks (day 35) after the first collection (day 0, baseline).

#### Species richness and diversity

**Fig 5:**
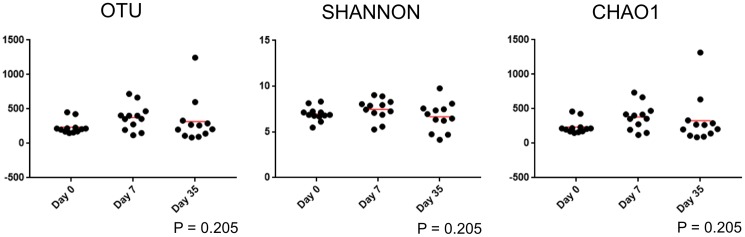
Scatter plots and statistical evaluation of 16S rRNA gene sequences obtained from 12 control eyes of 12 healthy horses at 3 timepoints: day 0, day 7, day 35. There is no difference in alpha diversity in control eyes over time (Friedman test and Dunn’s post-test).

There was no significant difference in alpha diversity in control eyes based on the sampling time point (Table 4 and Fig 5).

**Table 4:** Summary of alpha diversity indices at a depth of 9,610 sequences per sample for control eyes over time.

Control Eyes	Day 0 (Baseline)	Day 7	Day 35	*P-value
**Observed OTUs**	235.1 ± 98.7	375.9 ± 184.0	319.9 ± 323.7	0.205
**Shannon**	7.0 ± 0.8	7.5 ± 1.1	6.7 ± 1.6	0.205
**Chao1**	236.0 ± 100.4	380.6 ± 187.5	329.6 ± 344.5	0.205

Values represent averages with standard deviations.

*P-values determined by Freidman test and Dunn’s post-test with significance level < 0.05.

#### Microbial community structure

**Fig 6:**
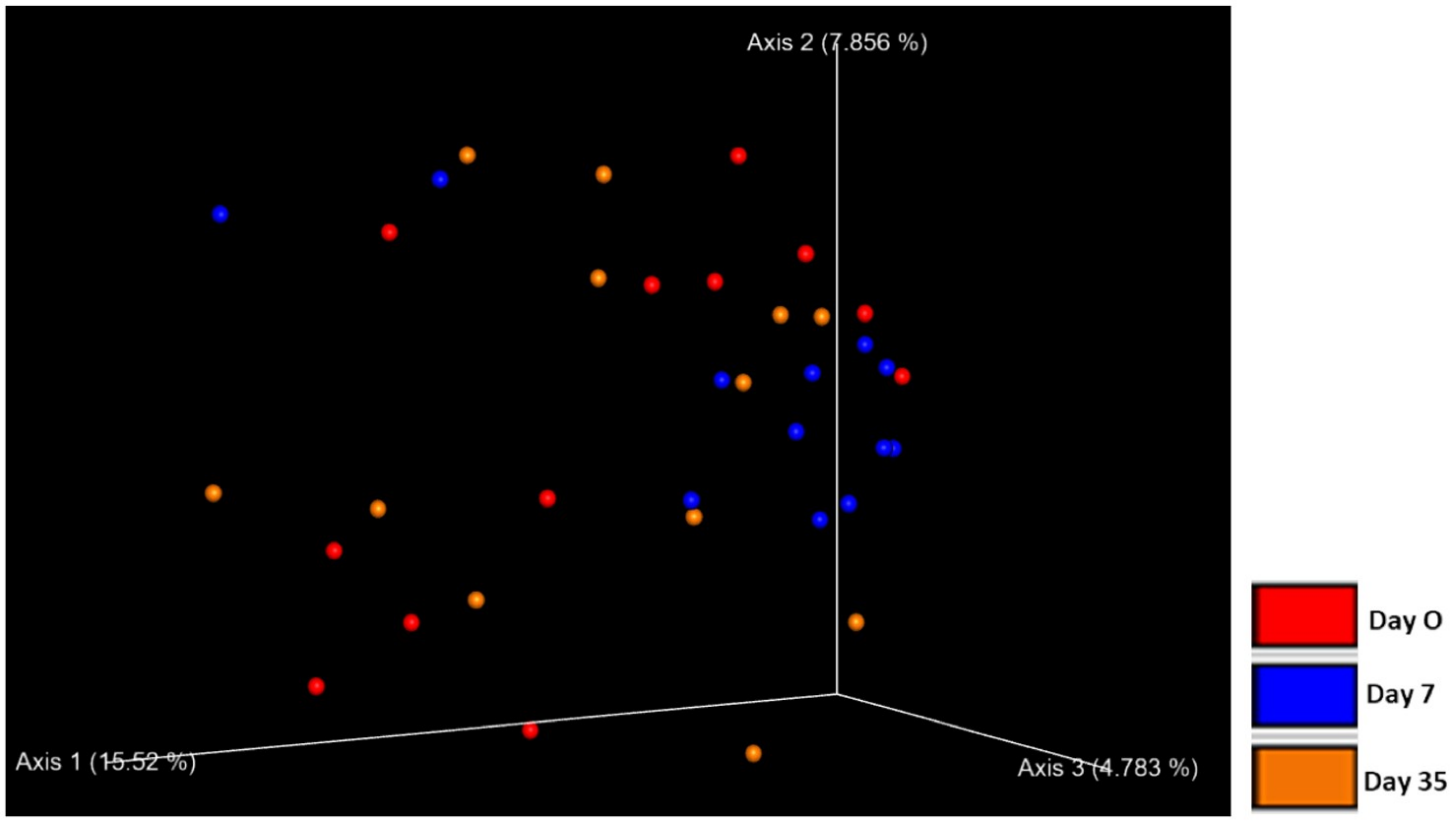
Principle coordinate analysis plot (PCoA) of unweighted UniFrac distance matrices of 12 control eyes from 12 healthy horses at three timepoints: day 0, day 7, day 35. No clustering was observed indicating there was no difference in beta diversity in control eyes over time.

There was no difference in beta diversity in control eyes over time as evident by the lack of clustering in the PCoA plot (Fig 6). No significant difference in microbial communities was observed with ANOSIM (weighted UniFrac, R = -0.005, R = -0.044, R = 0.023 for day 0 vs. 7, day 0 vs. 35, and day 7 vs. 35, respectively, p > 0.05); (unweighted UniFrac, R = 0.088, R = -0.015, R = 0.073 for day 0 vs. 7, day 0 vs. 35, and day 7 vs. 35, respectively, p > 0.05).

#### Microbial community composition

**Fig 7:**
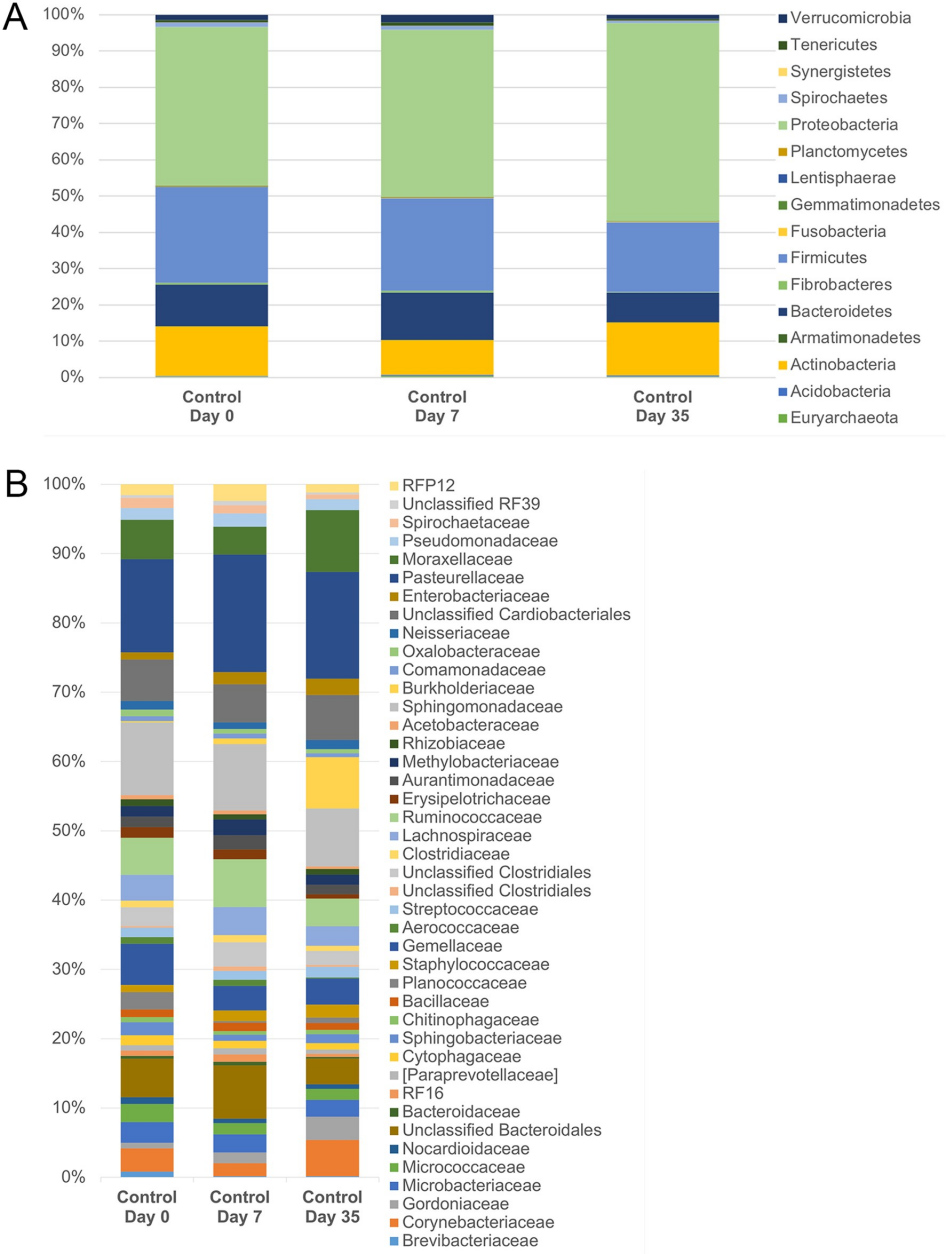
Temporal composition by bacterial phyla (A) and families (B) in control eyes. Data are presented at baseline (day 0), day 7, and day 35. The bars represent mean percentage of sequences, totaling 100% at each time point.

Fig 7 shows the relative abundance of bacteria in control eyes sampled over time. Using Friedman and Dunn’s multiple comparison tests, we discovered select bacteria were differentially abundant on the ocular surface of control eyes over time (Table 5). No significant changes were detected at the phylum or family level. At the genus level, control eyes had significantly more *Brevibacterium* on day 0 compared to day 7 and day 35 (p = 0.02, q = 0.049). *Burkholderia* was significantly enriched in control eyes on day 35, compared to day 0 and day 7 (p < 0.001, q = 0.028).

**Table 5:** Temporal variation of bacterial genera isolated from the ocular surface of control eyes of healthy horses at three time points. Median relative percentages and ranges of bacterial groups, annotated to level of phylum, family and genus, based on sequencing of 16S rRNA.

Taxon	Day 0		Day 7		Day 35			
Phylum Family *Genus*	Median *%	Range %	Median %	Range %	Median %	Range %	P-value **	Q-value ***
**Proteobacteria**	46.2	14.5–74.1	43.2	17.9–80.4	51.5	27.9–93.2	0.558	0.670
*Acinetobacter*	1.9^a^	0–5.9	0.7^a^^,^^b^	0.1–2.5	0.4^b^	0–2.2	**0.017**	0.226
*Burkholderia*	0^a^	0–0.2	0^a^	0–5.3	0.6^b^	0–34	**<0.001**	**0.028**
Unclassified Enterobacteriaceae	0^a^	0–1.4	0.3^a^	0–2.3	0.5^a^	0–2.6	**0.037**	0.273
Unclassified Acetobacteraceae	0^a^	0–0.5	0.2^a^	0–0.5	0^a^	0–0.3	**0.045**	0.0283
**Firmicutes**	27.0	7.3–44.1	28.7	6.8–42.7	21.1	1.7–32.9	0.124	0.397
Unclassified Ruminococcaceae	1.7^a^^,^^b^	0.2–10.8	4.2^a^	0.2–11.9	1.8^b^	0–8.6	**0.028**	0.245
[Mogibacteriaceae]	0.6^a^^,^^b^	0–2.2	0.8^a^	0–2.4	0.3^b^	0–1.5	**0.002**	0.064
Unclassified Bacillaceae	0.1^a^	0–1.9	0.3^a^	0–1.7	0^a^	0–0.9	**0.040**	0.281
*Facklamia*	0^a^	0–0.6	0.2^a^	0–0.5	0^a^	0–0.3	**0.035**	0.273
*Coprococcus*	0^a^	0–0.6	0.2^a^	0–1	0^a^	0–0.8	**0.048**	0.283
**Actinobacteria**	9.8	3.1–42.4	10.1	3.5–19	13.6	3.6–41.9	0.264	0.431
*Brevibacterium*	0.2^a^	0–2.7	0^b^	0–0.4	0^b^	0–0.9	**0.001**	**0.049**
**Bacteroidetes**	8.9	5.6–25.6	14.4	2.2–25.2	7	0.8–18.5	0.264	0.431
Unclassified BS11	0.1^a^	0–1.3	0.4^b^	0–1.5	0.1^a^	0–0.6	**0.003**	0.091
*BF311*	0.2^a^	0–1.2	0.2^a^	0–0.7	0^a^	0–0.6	**0.020**	0.237

*^a,b^: Median values not sharing a common superscript differ significantly (p < 0.05, Dunn’s multiple comparison post-test).

**: P-values based on the Friedman test

***: Q-values adjusted based on the Benjamini & Hochberg False discovery rate

Based on LEfSe analysis, *Brevibacterium* was increased on day 0 and *Burkholderia* was increased on day 35 among control eyes (Table 6). LEfSe also demonstrated differences in relative taxa abundance of select bacterial families and genera over time. For example, an unclassified family of BS11 was increased on day 7.

**Table 6:** Linear discriminant analysis of bacterial taxa in control eyes and their associations with each time point. Only LDA scores of >3.0 are shown.

Taxa	LDA	Time point
Family		
Brevibacteriaceae	3.45	Day 0
BS11	3.29	Day 7
Burkholderiaceae	4.45	Day 35
*Genus*		
*Brevibacterium*	3.45	Day 0
Unclassified BS11	3.22	Day 7
*Burkholderia*	4.45	Day 35

### Temporal variability of ocular surface microbiome in eyes treated with neomycin-polymyxin-bacitracin

In order to understand the temporal stability of the ocular surface microbiome in healthy horses following topical antibiotic use, two additional samples were collected from treatment eyes following baseline (day 0). This occurred after one week of antibiotic therapy was applied to the eye three times daily (day 7), and four weeks after discontinuing antibiotic therapy (day 35).

#### Species richness and diversity

**Fig 8:**
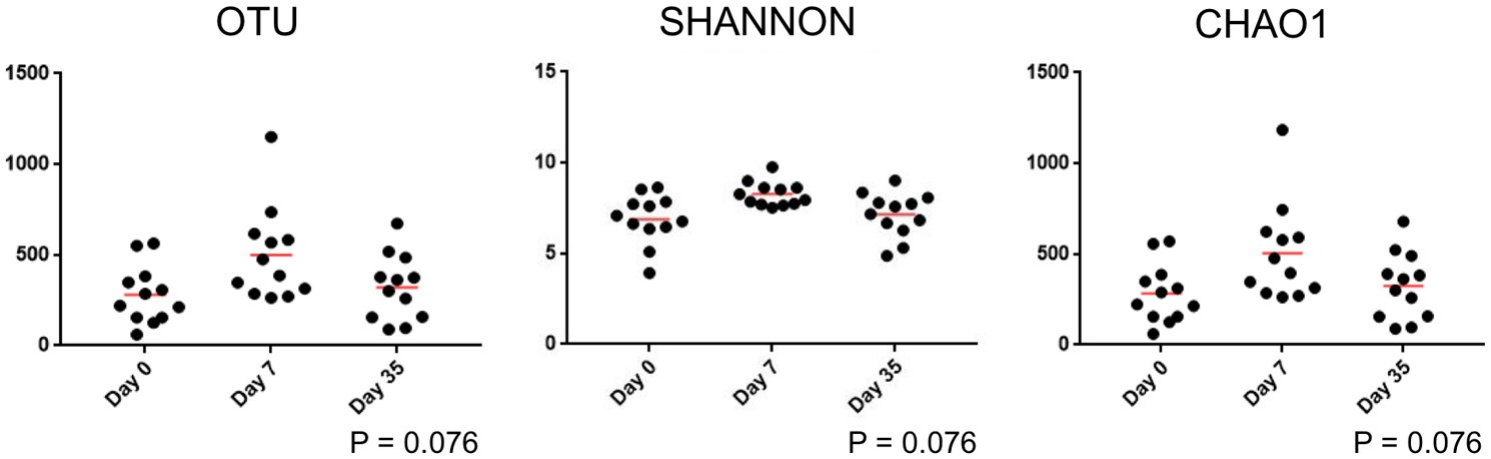
Scatter plots and statistical evaluation of 16S-rRNA gene sequences obtained from 12 treatment eyes of 12 healthy horses at 3 timepoints: Baseline (day 0), after one week of topical antibiotic therapy (day 7), four weeks after discontinued topical antibiotic therapy (day 35). There is no difference in alpha diversity in treated eyes over time (Freidman test and Dunn’s post-test).

There was no significant difference in alpha diversity in treatment eyes based on the sampling time point (Table 7 and Fig 8).

**Table 7:** Summary of alpha diversity indices at a depth of 9,610 sequences per sample for treatment eyes over time.

Treatment Eyes	Day 0 (Baseline)	Day 7	Day 35	*P-value
**Observed OTUs**	283.2 ± 152.0	506.2 ± 266.4	324.2 ± 183.5	0.076
**Shannon**	6.9 ± 1.4	8.3 ± 0.7	7.1 ± 1.2	0.076
**Chao1**	283.2 ± 162.0	506.2 ± 266.4	324.2 ± 183.5	0.076

Values represent averages with standard deviations.

*P-values determined by Freidman test and Dunn’s post-test with significance level < 0.05.

#### Microbial community structure

**Fig 9:**
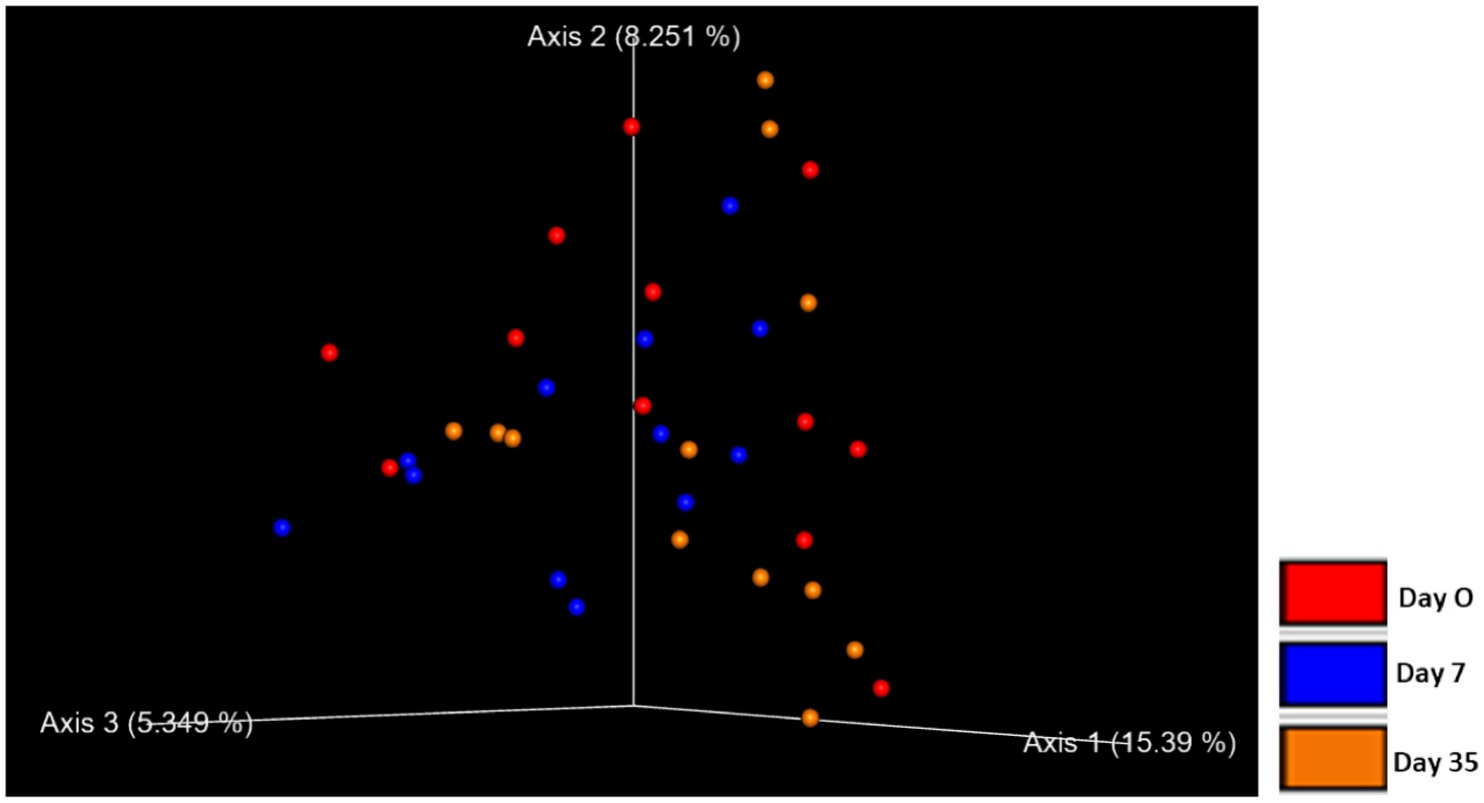
Principle coordinate analysis plot (PCoA) of unweighted UniFrac distance matrices of 12 treatment eyes from 12 healthy horses at three timepoints: Baseline (day 0), after one week of topical antibiotic therapy (day 7), four weeks after discontinued topical antibiotic therapy (day 35). No clustering was observed indicating there was no difference in beta diversity in treatment eyes over time.

There was no difference in beta diversity in treatment eyes over time as evident by the lack of clustering in the PCoA plot (Fig 9). No significant difference in microbial communities was observed with ANOSIM (weighted UniFrac, R = 0.176, R = -0.011, R = 0.162 for day 0 vs. 7, day 0 vs. 35, and day 7 vs. 35, respectively, p > 0.05); (unweighted UniFrac, R = 0.1, R = -0.038, R = 0.085 for day 0 vs. 7, day 0 vs. 35, and day 7 vs. 35, respectively, p > 0.05).

#### Microbial community composition

**Fig 10:**
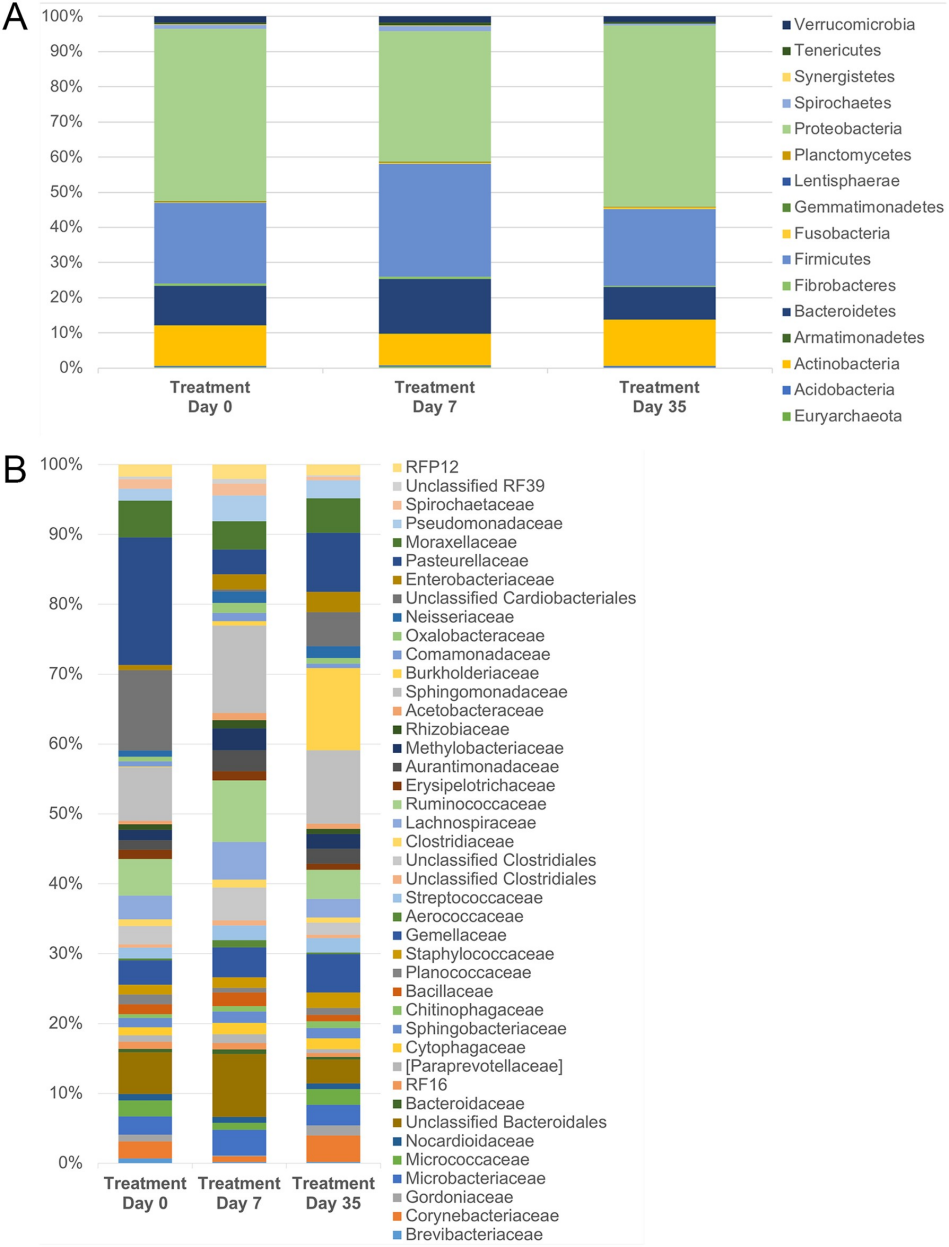
Temporal composition by bacterial phyla (A) and families (B) in treated eyes. Data are presented at baseline (day 0), day 7, and day 35. The bars represent mean percentage of sequences, totaling 100% at each time point.

Fig 10 shows the relative abundance of bacteria from treatment eyes sampled over time. Using Friedman and Dunn’s multiple comparison tests, we discovered a trend for select bacteria to be differentially abundant on the ocular surface of treatment eyes over time (Table 8). When p-values were corrected for false discovery rate, however, no significant changes were detected at the phylum, family, or genus level (q > 0.05).

**Table 8:** Temporal variation of bacterial genera isolated from the ocular surface of treatment eyes of healthy horses at three time points. Median relative percentages and ranges of bacterial groups, annotated to level of phylum, family and genus, based on sequencing of 16S rRNA.

Taxon	Day 0		Day 7		Day 35			
Phylum Family *Genus*	Median *%	Range %	Median %	Range %	Median %	Range %	P-value **	Q-value ***
**Proteobacteria**	45.6	11.9–94	36.6	15.3–55.5	52.9	29.2–77.2	0.338	0.469
*Sphingomonas*	5^a^	0.6–17.5	9.4^a^	3.2–17.5	8.1	0.7–16	**0.028**	0.167
Unclassified Pasteurellaceae	8.7^a^^,^^b^	1–72.3	0.8^a^	0.1–5.9	0.7^b^	0–55.9	**0.006**	0.096
*Actinobacillus*	0^a^	0–2	0.7^a^	0–6.7	0.4^a^	0–3.2	**0.048**	0.2207
*Acinetobacter*	1.8^a^	0.2–8.3	0.7	0.2–9.5	0.9^a^	0–4.6	**0.039**	0.191
Unclassified Aurantimonadaceae	0.8^a^	0–3.5	1.9^a^	0.8–5.1	1.3	0–3.5	**0.006**	0.096
*Methylobacterium*	0.6^a^	0–3.3	1.6^a^	0.8–4.4	1.2	0.3–4.6	**0.013**	0.115
Unclassified Enterobacteriaceae	0.1^a^^,^^b^	0–1.1	0.8^a^	0.2–2	1^b^	0–4.6	**0.001**	0.094
Unclassified Oxalobacteraceae	0.3^a^	0–1.2	0.7^a^	0.1–2.7	0.6	0.2–1	**0.004**	0.094
Unclassified Cardiobacteriales	0.5^a^^,^^b^	0.2–88.8	0.3^a^	0–0.4	0.1^b^	0–48.2	**0.002**	0.094
Unclassified Comamonadaceae	0^a^	0–1.3	0.5^a^	0–2.2	0.2	0–0.4	**0.012**	0.112
*Burkholderia*	0^a^	0–0.1	0	0–2.8	0.5^a^	0–53.8	**0.001**	0.094
Unclassified Rhizobiaceae	0^a^	0–0.3	0.3^a^	0.1–0.7	0	0–0.4	**0.006**	0.096
Unclassified Sphingomonadaceae	0^a^	0–0	0.2^a^	0–0.5	0	0–0.6	**0.012**	0.112
*Kaistobacter*	0^a^	0–0.8	0.2^a^	0–0.9	0.2^a^	0–1.4	**0.036**	0.189
**Firmicutes**	20	2–44.2	31.4	17.5–45.2	19	1.7–41.1	0.205	0.336
Unclassified Ruminococcaceae	3.1	0–12.3	5.2^a^	2.3–13.1	2.6^a^	0–8.2	**0.028**	0.167
Unclassified Clostridiales	1.8	0–7.9	4^a^	1.3–7.9	1.3^a^	0–4.2	**0.009**	0.102
Unclassified [Mogibacteriaceae]	0.3	0–1.6	0.7^a^	0.2–1.5	0.2^a^	0–1.1	**0.004**	0.094
*Clostridium*	0.4	0–1.5	0.5^a^	0.2–1.5	0.3^a^	0–1.2	**0.011**	0.112
Unclassified Bacillaceae	0^a^	0–0.2	0.3^a^	0.1–1	0^a^	0–0.7	**0.003**	0.094
*Facklamia*	0^a^	0–0.6	0.3^a^	0.1–1	0^a^	0.0.5	**0.028**	0.166
*Phascolarctobacterium*	0.1	0–0.7	0.3^a^	0.1–0.7	0^a^	0–0.5	**0.009**	0.102
*p-75-a5*	0.2	0–0.7	0.2^a^	0–0.9	0.1^a^	0–0.2	**0.029**	0.166
**Actinobacteria**	9.7	1.4–25.4	9.2	4.7–14.4	10.4	5–27	0.174	0.336
*Corynebacterium*	1.1	0.3–5.8	0.7^a^	0.2–1.3	1.3^a^	0.5–22.9	**0.036**	0.189
*Gordonia*	0.6	0–2.5	0^a^	0–0.4	0.8^a^	0–3.9	**0.004**	0.094
Unclassified Pseudonocardiaceae	0.2^a^	0–0.8	0.3^a^	0–0.9	0.2	0–0.9	**0.020**	0.156
Unclassified Geodermatophilaceae	0^a^	0–0.2	0.2^a^	0.1–0.7	0.2	0–0.4	**0.024**	0.166
**Bacteroidetes**	9.1	0.9–26.4	15	9.7–22.7	9.1	2–18.5	0.075	0.226
Unclassified RF16	0.9^a^	0–2.7	0.8	0.3–1.6	0.4^a^	0–1.7	**0.017**	0.134
Unclassified Sphingobacteriaceae	0.2^a^	0–1.7	0.9^a^	0.4–2	0.6	0–2.3	**0.017**	0.134
*BF311*	0.2	0–0.8	0.3^a^	0.1–0.8	0.1^a^	0–0.6	**0.044**	0.203

*^a,b^: Median values not sharing a common superscript differ significantly (p < 0.05, Dunn’s multiple comparison post-test).

**: P-values based on the Friedman test

***: Q-values adjusted based on the Benjamini & Hochberg False discovery rate

LEfSe analysis demonstrated differences in relative taxa abundance of select bacterial families and genera over time (Table 9). BS11, Acetobacteraceae, Ruminococcaceae, and an unclassified order of Clostridiales were amongst bacteria increased on the ocular surface of treatment eyes on day 7, while Enterobacteriaceae, Corynebacteriaceae, and Burkholderiaceae were enriched on day 35 (Table 9 and Fig 10).

**Table 9:** Linear discriminant analysis of bacterial genera in treatment eyes and their associations with each time point. Only LDA scores of >3.0 are shown.

Taxa	LDA	Time point
**Phylum**		
**Bacteroidetes**	4.39	Day 7
**Proteobacteria**	4.93	Day 35
Family		
Porphyromonadaceae	3.19	Day 7
BS11	3.22	Day 7
Acetobacteraceae	3.23	Day 7
Mogibacteriaceae	3.38	Day 7
Methylobacteriaceae	3.67	Day 7
Ruminococcaceae	4.21	Day 7
Unclassified Clostridiales	4.27	Day 7
Gordoniaceae	3.70	Day 35
Enterobacteriaceae	3.71	Day 35
Corynebacteriaceae	4.12	Day 35
Burkholderiaceae	4.59	Day 35
*Genus*		
-C*oprococcus*	3.10	Day 7
-Unclassified BS11	3.16	Day 7
-Unclassified Mogibacteriaceae	3.19	Day 7
-Unclassified Methylobacteriaceae	3.27	Day 7
-Unclassified Comamonadaceae	3.36	Day 7
-Unclassified Oxalobacteraceae	3.38	Day 7
-Unclassified Lachnospiraceae	3.87	Day 7
-Unclassified Clostridiales	3.96	Day 7
-Unclassified Ruminococcaceae	4.02	Day 7
-Unclassified Bacteroidales	4.24	Day 7
*-Rothia*	3.26	Day 35
*-Gordonia*	3.68	Day 35
*-Corynebacterium*	4.14	Day 35
*-Burkholderia*	4.56	Day 35

## Discussion

The present study demonstrates the equine ocular surface contains a more diverse bacterial community than previously detected using standard culture techniques. A total of 5 bacterial phyla and 10 families were present in all 24 eyes at >1% relative abundance (Table 3). The most common phyla and their relative proportions colonizing the equine ocular surface, Proteobacteria (46.1%), Firmicutes (24.6%), Actinobacteria (12.6%), and Bacteroidetes (11.2%), are comparable to investigations of the human ocular surface [14,15,17]. Preliminary studies describing the ocular surface microbiome of companion animals utilizing NGS identified the same four phyla at different proportions, with Firmicutes most abundant across all canine and feline samples (34.9% and 43%, respectively) [18,19]. Direct comparison of microbiome studies; however, should be interpreted with caution as there exist variations in methodologies for DNA extraction, sequencing, analysis, and clustering strategies.

The most relatively abundant bacterial families sequenced from all 24 equine eyes were Pasteurellaceae (13.7%), Sphingomonadaceae (7.9%), and an unclassified order of Cardiobacteriales (7.7%). Other commonly identified families present in 96–100% of eyes included an unclassified order of Bacteroidales (5%), Moraxellaceae (4.8%), Ruminococcaceae (4.5%), and Gamellaceae (4.1%). The majority of the most relatively abundant microorganisms are gram-negative, which contradicts the previous notion of a gram-positive dominant microbiota. In fact, gram-positive bacteria commonly cultured from the equine ocular surface, such as *Corynebacterium*, *Streptococcus*, *Staphylococcus*, and *Bacillus spp*., were present in 63–92% of eyes and represented 2.6%, 1.3%, 1.0% and 1.0% of the bacterial genera sequenced, respectively (Table 3).

Several taxa isolated via NGS methods in this study were never before associated with the equine ocular surface, likely due to their inability to grow in the laboratory despite a wide selection of agars, media, and culture techniques. This includes families from the phyla Proteobacteria (unclassified order of Cardiobacteriales, Aurantimonadaceae, Methylobacteriaceae, Rhizobiaceae, Comamonadaceae), Firmicutes (Ruminococcaceae, Gemellaceae, Lachnospiraceae, an unclassified order of Clostridiales, Planococcaceae, Erysipelotrichaceae, Clostridiaceae), Actinobacteria (Microbacteriaceae, Brevibacteriaceae, Gordoniaceae, Nocardioidaceae), Bacteroidetes (unclassified order of Bacteroidales, Sphingobacteriaceae, Cytophagaceae, RF16, [Paraprevotellaceae]), Verrucomicrobia (RFP12), and Spirochaetes (Spirochaetaceae) (Table 3). Although advancements in technology have allowed us to detect these taxa, we currently have a limited understanding of their impact on the health of the equine ocular surface.

There were no significant differences in either alpha or beta diversity among control eyes when sampled at three separate time points: day 0, day 7, and day 35. Additionally, there was no significant difference in the relative abundance of bacterial phyla or families over time. This finding supports the notion that the ocular surface microbiome maintains stability with regard to species richness, community structure, and community composition over time. The temporal stability identified in this study supports the likely presence of a core ocular surface microbiome in equine eyes.

Despite several NGS studies in physician ophthalmology, there remains no consensus on whether the human ocular surface microbiome constitutes a core or transient community [5,6,13–17,27]. The ocular surface is an open system constantly exposed to the environment and, as a consequence, myriad microorganisms. Compared to other open systems such as the skin, nasal cavity, and oral cavity, the ocular surface contains a relatively low microbial biomass [5,13,16,17]. This is likely due to innate defense mechanisms that protect the ocular surface from infection by pathogenic organisms. In conjunction with blinking and tearing, the tear film contains a plethora of antimicrobial properties [28]. Therefore, the ocular surface may be less conducive to the establishment of a stable core microbiome, and more supportive of a variation of transient microbes. The ocular surface bacterial community is distinct in comparison to other regions of the body, such as the skin and oral cavity [16]. Thus, the ocular surface microbiome is not simply an extension of the skin’s microbiome nor likely solely comprised of transient microbes.

Results of the current study support the presence of both a core and transient microbiome on the equine ocular surface. The majority of identified bacterial taxa were present in all eyes at every time point sampled, consistent with a stable core community of microbes. However, there was apparent variation in the relative abundances of taxa both between eyes and between individual horses at baseline. Furthermore, the relative abundance of two select genera, *Brevibacterium* and *Burkholderia*, altered significantly over time in control eyes. These minor variations likely represent the components of a transient microbiome that is influenced both by external factors in the environment and the host’s innate immune defenses.

There were no significant differences in either alpha or beta diversity among treatment eyes when sampled at baseline (day 0), after one week of antibiotic therapy with neomycin-polymyxin-bacitracin (day 7), and four weeks after discontinuing antibiotic therapy (day 35). Additionally, there was no significant difference in the relative abundance of bacterial phyla, families, or genera in treatment eyes over time. These findings support the notion that, despite a short-term course of broad-spectrum topical antibiotics, the ocular surface microbiome maintains stability with regard to species richness, community structure, and community composition.

Topical antibiotics therapy is thought to alter microbial populations on the ocular surface. A prospective controlled culture-based study evaluating topical neomycin-polymyxin-bacitracin application to healthy horse eyes found a transient reduction in positive bacterial cultures after one week of treatment, with a repopulation to pre-treatment numbers after two weeks of treatment [10]. Although the current study detected both increasing and decreasing trends in the relative abundance of various bacterial families and genera in treatment eyes over time (Table 8), the results were not significant and more likely influenced by multiple environmental and host factors rather than antibiotic treatment.

Chronic use of topical ophthalmic antibiotics for several weeks to months may have more profound effects on the microbial composition of the ocular surface, and facilitate the emergence of resistant strains [7,29,30]. Furthermore, horses are prone to developing serious, vision-threatening ocular infections in the form of ulcerative keratitis or corneal abscessation, which are often treated with a prolonged course of frequently applied topical antimicrobials [1–4]. Therefore, future investigations are warranted to evaluate the effects of chronic antimicrobial use on the ocular surface microbiome using NGS.

There are several limitations to this study including a relatively small and heterogeneous study population. We aimed to obtain as much homogeneity as possible with regard to housing, diet, and patient signalment, while also representing the general equine population. It is thus conceivable that other equine populations might demonstrate different resident microbial populations. Currently, there is paucity in the literature regarding the effect of population variations such as age, sex, season, geography, and other environmental factors on the composition of the ocular surface microbiome. This, together with intrinsic variation noted between eyes and individuals in current and previous studies [16,17], challenges how we interpret the significance of microbiome data. Such considerations warrant further investigation with larger scale studies to limit bias.

Another limitation in the interpretation of microbiome studies is the evaluation of relative abundance, which does not represent the absolute quantities of the microbial populations present [31]. Absolute abundance is difficult to obtain from NGS; however, quantitative PCR of specific organisms can be performed and considered with future studies to determine absolute quantities of a known sequence in a sample. Lastly, NGS detects the presence of DNA of a particular organism but cannot elucidate whether or not it came from a viable population, or simply succumbed to the eye’s natural defense mechanisms upon contact with the ocular surface [13]. Despite the inherent limitations of NGS, there remains an abundance of useful data that, in conjunction with future studies, will undoubtedly enhance our understanding of the role of the ocular surface microbiome in health and disease.

## Conclusion

This is the first report to investigate the bacterial community of the healthy equine ocular surface using molecular-based techniques. This is also the first report to examine the temporal stability of the equine ocular surface microbiome both over time and following topical antibiotic therapy. Richer and more diverse microbial communities inhabit the ocular surface of the equine eye than previously detected with conventional culture techniques. A stable core bacterial microbiome was identified and discovered to remain consistent over time and with short-term topical broad-spectrum antibiotic use. Investigations comparing the equine ocular surface microbiome of healthy and diseased eyes are currently underway to determine if alterations to ocular microbial communities are associated with disease.
